# OctoPartenopin: Identification and Preliminary Characterization of a Novel Antimicrobial Peptide from the Suckers of *Octopus vulgaris*

**DOI:** 10.3390/md18080380

**Published:** 2020-07-23

**Authors:** Valeria Maselli, Emilia Galdiero, Anna Maria Salzano, Andrea Scaloni, Angela Maione, Annarita Falanga, Daniele Naviglio, Marco Guida, Anna Di Cosmo, Stefania Galdiero

**Affiliations:** 1 Department of Biology, University of Naples “Federico II”, via Cinthia, 80126 Naples, Italy; valeria.maselli@unina.it (V.M.); emilia.galdiero@unina.it (E.G.); angela.maione3@gmail.com (A.M.); marco.guida@unina.it (M.G.); 2 Proteomics & Mass Spectrometry Laboratory, ISPAAM, National Research Council, 80147 Naples, Italy; annamaria.salzano@cnr.it (A.M.S.); andrea.scaloni@ispaam.cnr.it (A.S.); 3 Department of Agricultural Science, University of Naples “Federico II”, Via dell’Università 100, 80055 Naples, Italy; annarita.falanga@unina.it; 4 Department of Chemical Sciences, University of Naples “Federico II”, via Cinthia, 80126 Naples, Italy; naviglio@unina.it; 5 Department of Pharmacy, University of Naples “Federico II”, Via Mezzocannone 16, 80134 Naples, Italy

**Keywords:** *Octopus vulgaris*, antimicrobial peptides, antimicrobial activity, antibiofilm activity

## Abstract

Microorganism resistance to conventional antibiotics represents one of the major global health concerns. This paper focuses on a peptide (OctoPartenopin) extracted from suckers of *Octopus vulgaris;* bioassay-guided chromatographic fractionation was used to identify this sequence, which holds significant antibacterial activity against Gram-positive and Gram-negative bacteria. OctoPartenopin is encrypted within the calponin sequence and was associated with the high levels of proteolytic activity already reported in octopus arm suckers. We synthesized the parent peptide and four analogues; all peptide were tested for their antibacterial and antibiofilm activities. Preliminary antibiofilm experiments showed that that one of the analogues had the best activity in both inhibition and eradication of biofilm of all three microorganisms tested. The occurrence of OctoPartenopin in arm suckers provided novel speculative information on animal behavior, as concerns maternal care of fertilized eggs. Our results highlight that suckers are a rich source of multifaceted peptides to develop alternative antimicrobial agents and food preservatives.

## 1. Introduction

Octopus is a highly versatile and opportunistic predator [1,2,3]; it hunts for food by ‘speculative pounce’ [4] and ‘groping’, and it explores the surfaces of rocks or the sea-bed with their arms, webs, and suckers [5]. The common octopus, *Octopus vulgaris* (Cuvier, 1797) is a cosmopolitan merobenthonic cephalopod with a short life cycle [6], living on rocky, sandy, and muddy bottoms from the coastline to the edge of the continental shelf, and it is very common in the Mediterranean Sea and eastern Atlantic Ocean [7]. Octopus and all cephalopods, as a result of evolutionary selective pressure, have developed a winning strategy for surviving in different environments [8,9]. *O. vulgaris* is an economically important seafood species [10], and a model for studying complex behavioral, neuronal, and genomic plasticity [11,12]. Moreover, it is well known for its intelligence linked to adult neurogenesis mechanisms, curiosity, and adaptability; recently, transcriptomic and genomic tools were developed to investigate these issues on this species and the congeneric *O. bimaculoides* [8,13,14,15]. Most octopus neurons are found in the arms, which can independently taste and touch, and control basic motions without input from the brain [16,17,18]. Each arm is made of a dozen of suckers and is packed with hundreds of sensors [19,20,21,22,23].

Interestingly, the octopus female leaves its eggs to fend for themselves, and eggs are resistant to infections. In particular, it is believed that cephalopod antimicrobial peptides (AMPs) are trapped in the egg capsule and mainly expressed by female accessory sex glands, conferring on them efficient protection of organs against microorganisms [24]. In this context, studies reporting the identification of AMPs in cephalopods were conducted on squids and cuttlefish [25,26]. Nevertheless, there is still a scarcity of knowledge regarding the defense mechanisms involved in *O. vulgaris* immune response, even if more researchers are focusing their attention on this topic [13,27,28,29,30,31]. As a matter of fact, octopus lacks an adaptive immune system [32,33], but has an efficient innate immune system comprised of cellular and humoral components that act as the first line of defense against a broad spectrum of pathogens [13,34]. Although the marine environment presents various and high levels of exposure to a considerable number of pathogens, only granulocytes were observed in the hemolymph of three species of Octopoda [35,36], and they were characterized and cultured in *O. vulgaris* [31,37]. At the same time, *O. vulgaris* presents a hemagglutination activity [38,39] and an anti-protease activity associated with α-macroglobulin in the hemolymph [40]. In order to fight against pathogens, it is likely that octopus exploits secreted AMPs as part of an innate defense mechanism, similarly to other aquatic animals [41,42,43,44,45,46].

The growing problem of resistance to conventional antibiotics and the need for novel drugs has stimulated interest in the development of antimicrobial peptides as human therapeutics (ADP, http://aps.unmc.edu/AP/main.php). Recently, much attention has been directed towards marine-derived bioactive peptides due to their special living environment, composition, and properties as well as to their antiviral, antitumor, antidiabetic, and antihypertensive activities and their role in the food industry for preservation and elongation of shelf-lives [47]. Since marine organisms live in close contact with microbes, they have proved to be a rich source of AMPs [48,49] with novel chemistry and diverse biological properties [47,50,51,52,53,54].

For example, AMPs were described in many mollusks as bivalves [55]; cysteine-rich peptides were identified in mussels [56]; defensins and proline-rich peptides were found in oysters [57] and gastropods [58,59]. Proteins with antimicrobial activity, such as egg case proteins Sep-ECPs [60] or hemocyanin [59,61], were identified in gastropods. Moreover, several other AMPs were identified encrypted within the sequence of proteins and could be derived from the cleavage of bulky proteins; interestingly, several AMPs from marine organisms used as food sources are released in the corresponding body fluids/tissues following the action of specific proteases or hydrolytic treatments [62], similarly to what has been observed in other animal products [63,64]. Due to their natural origin and antimicrobial activity, the latter molecules were proposed as additives in food industry for the preservation of edible products and the elongation of corresponding shelf-life [53], but also as active ingredients for the preparation of packaging materials.

The pharmaceutical company interest in AMPs is linked to their ability to disrupt bacterial membranes and, following cell internalization, to target components, such as nucleic acids, preventing microorganisms from developing resistance. Their main molecular features are a predominant cationic nature and a high percentage of hydrophobic residues, which enable them to assume an amphipathic primary or secondary structure. The low propensity to induce resistance, coupled with their low toxicity and the possibility of improving their activity through the rational design of peptidomimetics—together with high production through genetically engineered bacteria, bioreactor, or green-synthesis alternatives—make AMPs highly attractive for commercial purposes. On the other hand, AMPs from natural sources have also been widely used in the preservation of foods and to increase the product shelf-life [65].

In this context, octopus species and their relative transcriptomes were recently screened in order to identify novel AMPs [66,67]. The present study describes the identification of a novel AMP (OctoPartenopin) extracted from suckers of *O. vulgaris* that is active against bacteria and yeast species. The amino acid sequence identified is part of a repeated motif in calponin-like proteins, involved in muscular contraction. OctoPartenopin was used as lead molecule for the rational design of novel compounds, thus allowing the synthesis of four analogues with improved antimicrobial and antibiofilm activity. We also suggest a possible role of OctoPartenopin in maternal care of fertilized eggs.

## 2. Results

### 2.1. Antimicrobial Activity of the Sucker Extract and Purified Fraction

Octopus suckers aqueous extract (SE) (1 mg/mL) was assessed for its antimicrobial activity by evaluating the diameter of the clear zone of growth inhibition of Gram positive bacterium *S. aureus*, Gram-negative bacterium *P. aeruginosa*, and a yeast *C. albicans*. A clear inhibition zone was observed in the case of *S. aureus* and *P. aeruginosa*, but no activity was found towards *C. albicans* (Table 1).

**Figure 1:**
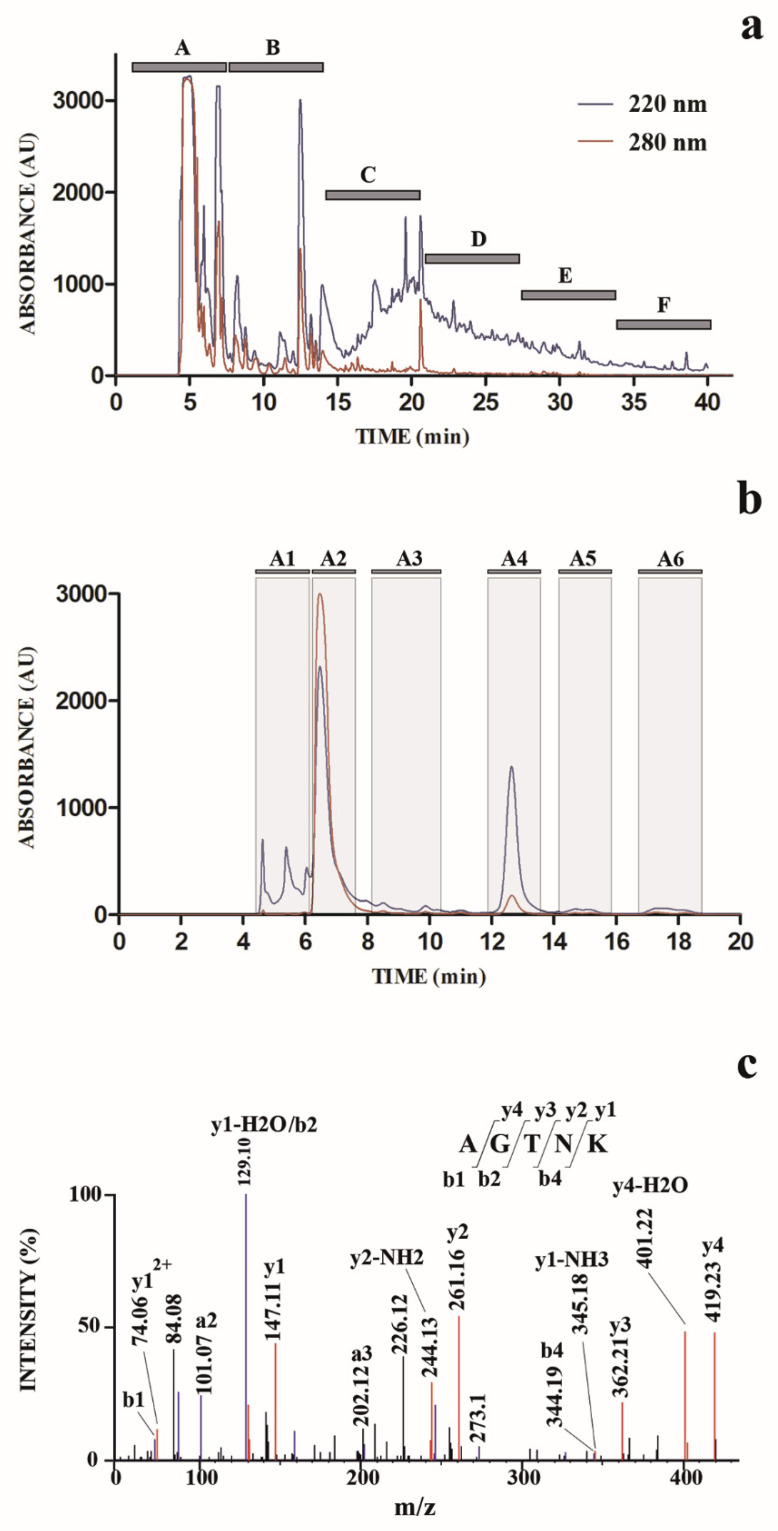
Natural peptide purification and characterization. (**a**) Reverse phase C18-HPLC separation of delipidated methanolic extract of octopus sucker (SE). Elution absorbance was measured at 220 nm (blue) and 280 nm (red). The eluate was collected to obtain fractions A–F. (**b**) Low-flow reverse phase C18-HPLC separation of delipidated methanolic extract of octopus sucker (SE). Elution absorbance was monitored at 220 nm (blue) and six fractions (A1–A6) were collected as indicated. (**c**) Fragmentation (MS/MS) spectrum of the doubly-charged ion at *m*/*z* 245.6348, which was acquired at 2.41 min during the nanoLC-ESI-Q-Orbitrap MS/MS analysis of fraction A4. Fragment ions of the b (blue) and y (red) series have been evidenced.

In order to isolate and identify molecular species responsible for the observed antimicrobial activity, SE was further subjected to a chromatographic fractionation on a semi-preparative reverse-phase C18 column (Figure 1a). Eluted fractions were collected on a time-based mode, to limit the number of fractions to analyze, and eluate was collected to finally obtain six fractions (A–F) (Figure 1a); aliquots (same volume) of each fraction were further subjected to the above-mentioned tests for antimicrobial activity. These assays evidenced that fraction A was the most active against the tested microorganisms (Table 1); thus, we concentrated our attention on it.

The chromatographic profile shown in Figure 1a suggested the presence in fraction A of a number of abundant hydrophilic compounds with similar retention times (eluting between 4 and 8 min); thus, an optimized chromatographic experiment was performed to separate and identify these species. Accordingly, SE fraction A was subjected to a dedicated semi-preparative chromatography in which the elution gradient as well as the flow rate (in this case lowered) were chosen with the aim to fractionate hydrophilic compounds. We collected six fractions (A1–A6) (Figure 1b) according to the recorded chromatographic profile; aliquots (same volume) of these fractions were further tested to determine the corresponding antimicrobial activity.

In vitro antimicrobial action of fractions A1–A6 was then evaluated by a broth microdilution method. Corresponding antimicrobial activity was expressed as minimum inhibitory concentration (MIC), results are reported in Table 2. All purified fractions were active against Gram-positive bacteria, Gram-negative bacteria, and fungi. Among the tested strains, the A1–A6 fractions showed the lowest activity against *C. albicans*, with MIC >300 µg/mL. Interestingly, another octopus peptide (octominin) exhibited a significant activity against this yeast, inducing cell wall damage and causing loss of cell membrane integrity [66,67].

All fractions showed a similar effect on *P. aeruginosa* and *S. aureus* even though the activity against *S. aureus* was more evident for fractions A2, A4, and A5 with a MIC of 120 µg/mL. In general, lower MIC values were obtained for the Gram-positive bacteria in comparison to the Gram-negative one (Table 2). Fraction A4 seemed to have the best antimicrobial activity against all the examined microorganisms, with MIC values of 120, 180, and 300 µg/mL for *S. aureus*, *P. aeruginosa*, and *C. albicans*, respectively. Accordingly, fraction A4 was selected for subsequent characterization.

### 2.2. Peptide Identification by Mass Spectrometry

The fraction A4 was selected for further structural characterization, which was accomplished by nanoLC-ESI-Q-Orbitrap MS/MS analysis. The use of a high-resolution mass spectrometer allowed us to measure the molecular mass values of the components present in this fraction with a high accuracy, together with those of the fragments originated during the MS/MS fragmentation analysis, thus allowing the identification of the corresponding molecular structure. Measured mass of mono- and multiply charged ions, corresponding isotopic distribution, and recorded mass values of resulting fragment ions strongly suggested the peptide nature of the molecules present in fraction A4. Thus, nanoLC-ESI-Q-Orbitrap MS/MS raw data were investigated by automated de novo peptide sequencing; results are reported in Supplementary Table S1. In order to guarantee the accuracy of the resulting data output, results were filtered to selectively maintain peptide sequence entries having a local confidence score for each amino acidic site >90%. Thus, pentapeptide AGTNK was identified as the molecular species with the largest area of ions extracted from the nanoLC-ESI-MS profile, whose sequence showed the highest average local confidence (ALC) score (>95%) (Supplementary Table S1). The other two identified amino acid sequences showed both lower areas of ions extracted from the nanoLC-ESI-MS profile, as well as were identified with lower ALC scores.

Identification of peptide AGTNK was confirmed by independent database searching of the nanoLC-ESI-Q-Orbitrap MS/MS raw data against the protein sequences of *O. bimaculoides*, which were retrieved from NCBI. In this case, database searching again identified the inner peptide AGTNK present in calponin-2-like isoform X1 (gi:961140629/NCBI reference sequence XP_014789315) as the molecular species showing the highest identification score. Several additional peptide sequences were also identified (Supplementary Table S1). Nevertheless, peptide AGTNK was largely more abundant than the other peptides, as demonstrated by the number of spectra (#Spec) corresponding to its sequence recorded during the nanoLC-ESI-Q-Orbitrap MS/MS analysis (Supplementary Table S1). The fragmentation MS/MS spectrum of the pentapeptide AGTNK is reported in Figure 1c; this compound was named OctoPartenopin.

### 2.3. MIC Determination of the Synthetized AGTNK Peptide

In order to confirm the above-mentioned results, and taking into consideration the importance of the C-terminal amide moiety in the enhancement of antimicrobial activity [68,69,70] and in the stabilization of peptide derivatives encrypted within the sequence of parental protein species [71], the peptide AGTNK was synthetized in its amide form yielding peptide P0, which was further purified and characterized for its antimicrobial activity (Table 3). For this synthetic product, MIC_80_ values of 150, 200, and 200 µM were measured in the case of *S. aureus*, *P. aeruginosa*, and *C. albicans*, respectively. These data indicated a higher activity of the pure synthetic compound in its amide form against *C. albicans*, when compared to the natural product present in the fraction A4.

### 2.4. Rational Design of Peptide Analogues

**Figure 2:**
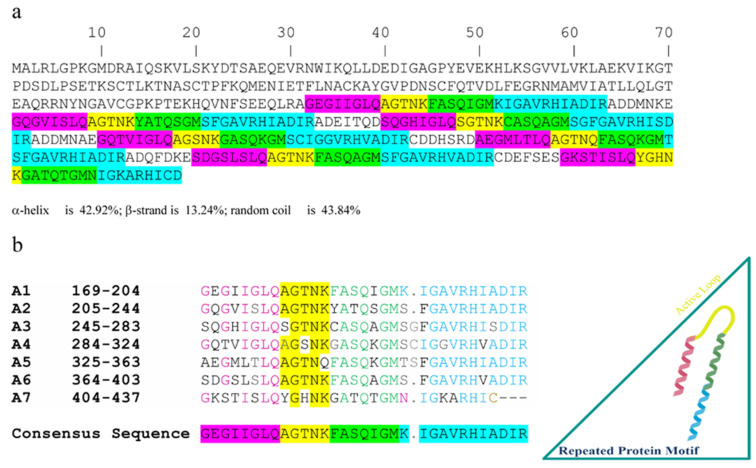
Sequence analysis of the pentapeptide AGTNK. (**a**) Sequence of calponin with the highlighted motif repeated seven times and contained the pentapeptide AGTNK homologs (reported in yellow). (**b**) Alignment of the seven repeating motifs (indicated with A1–A7) with the consensus sequence corresponding to peptide P4. Cartoon of secondary structure prediction of the consensus sequence P4 reported in the triangle box.

For rational design of analogues, we further analyzed and searched for the presence of the pentapeptide AGTNK in *Octopus* sp. Species. Homology searches of this sequence were performed with BLAST software on the NCBI web page (https://blast.ncbi.nlm.nih.gov/Blast.cgi), restricting the analysis only to *Octopus* sp. species. We found that the AGTNK sequence was present in proteins involved in muscular contraction, such as calponin isoforms identified in *O. bimaculoides* (XP_014789315), confirming mass spectrometry analysis results. Calponin protein family are conserved actin-binding components that, together with caldesmon, act as a thin-filament-based regulatory machinery in invertebrate smooth muscle contraction [72]. Calponin homologs were also found in the *O. bimaculoides* genome with more than 10 predicted alternative transcripts (Ocbimv22019629m.g) [14]. We focused our study on the analysis of the sequence of calponin-2-like isoform X1 (XP_014789315), which clearly revealed that the AGTNK sequence is present three times, while the A(S)GT(S)NK(Q) consensus is repeated seven times (Figure 2a).

In particular, the latter consensus sequence (reported in yellow in Figure 2) is part of a larger motif that is repeated seven times in the calponin primary structure (named A1–A7 in Figure 2b); these seven motifs are separated by a non-conserved sequence made of six amino acids. Furthermore, we performed a prediction of secondary structure of the protein calponin with several programs available on the web, such as GOR method [73] and YASSPP [74], and we found that the peptide AGTNK likely occurs in a loop region comprised between two α-helical domains.

The analysis of the tandem repeat sequence clearly shows the presence of a high content of hydrophobic residues and a total net charge of +2; moreover, when in helical structure, 11 hydrophobic residues are likely located on the same face, which is a typical feature of peptides able to perform antibacterial activities.

The alignment of the abovementioned large seven tandem repeats present in calponin-2-like isoform X1 showed that their amino acid sequence is highly conserved and mutations usually are characterized by a conservation of the features of the amino acid side chains (Figure 2b). The results of the mass analysis showed the presence not only of AGTNK, but also of the sequence QAGTNK; the alignment shows that the glutamine residue (Q) is highly conserved at peptide *N*-terminus and thus we decided to synthetize this sequence also, which was named P1.

Based on considerations reported above, underlining the possible importance of the seven large motifs present in calponin isoforms for the eventual production of several bioactive molecules from a single parental species, we decided to probe additional longer peptide sequences (compared to AGTNK), which were synthesized in their amide form at the molecular C-terminus and further assayed for antimicrobial activity.

In particular, we produced compound analogues containing: (i) the conserved Q residue at peptide *N*-terminus (compound P1); (ii) the conserved Q residue at peptide *N*-terminus plus the conserved motif of seven amino acids at the C-terminus (compound P2); (iii) the conserved motif of nine amino acids at peptide *N*-terminus (compound P3); (iv) the conserved motif of nine amino acids at peptide *N*-terminus plus two conserved portions of 7 and 12 amino acids at the C-terminus, corresponding to the whole tandem repeat region repeated seven times in the calponin sequence (compound P4) (Figure 2b; Table 3).

### 2.5. Antimicrobial Activity of the Peptide Analogues

Above-mentioned compounds were assayed in parallel for their antimicrobial activity. All peptide analogues showed enhanced activity against both Gram-positive and Gram-negative bacteria, and yeast, when compared to the parent peptide P0 (Table 3)**.** With respect to the latter compound, the addition of the Q residue at the *N*-terminus did not produce any significant enhancement of the activity. Interestingly, both analogues P2 and P3 displayed MIC values between 50 to 100 µM against Gram-positive and Gram-negative as well as yeast, with peptide P2 showing the best activity. The analogue P4 showed a similar activity to P3 against bacteria, but had a lower activity against *C. albicans*. In conclusion, longer sequences showed a greater activity than compound P0, and the peptide P2 elongated at the C-terminus had the best activity. On the contrary, the two analogues P3 and P4 elongated at the *N*-terminus presented a higher activity compared to the parent peptide (P0) but lower than P2.

### 2.6. Biofilm Inhibition and Eradication Assay

**Figure 3:**
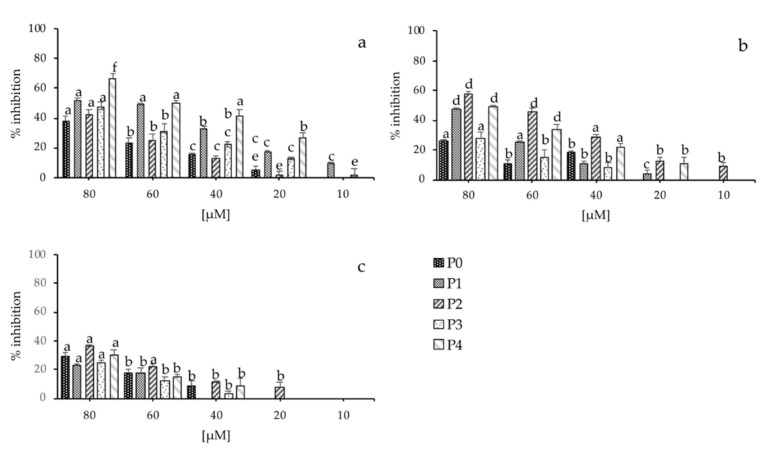
Inhibition activity of the synthetized peptides. Action of peptides P0, P1, P2 P3, and P4 on inhibition of microbial biofilm (**a**) *S. aureus*; (**b**) *P. aeruginosa*; (**c**) *C. albicans*. Error bars represent standard deviation. Different letters on top of each column (a–f) represent the significance at the 0.05 level (Tukey’s, *p* < 0.05).

It is known that microbial cells embedded in biofilm can increase resistance to antimicrobials compared to planktonic forms. The effects of peptide P0 and its analogues on biofilm formation and eradication of *S. aureus*, *P. aeruginosa*, and *C. albicans*—all strong biofilm producers—were investigated. Initially, we tested the inhibition activities of compound P0 and the four analogues. As shown in Figure 3, all of them significantly reduced biofilm formations of bacteria and fungi. A progressive inhibition of the biofilm formation was shown at concentration of 80 μM for peptides P1, P2, and P4, reaching an inhibition of about 60%, 60%, and 40% of *S. aureus*, *P. aeruginosa*, and *C. albicans*, respectively; while compound P0 and P3 reached less than 50% inhibition for all the strains tested at the same concentration. The inhibition rate decreased in a dose-dependent manner; in fact, at 10 μM the lowest concentration tested, peptides P1 and P3 inhibited about 10% of biofilm formation of *S. aureus*, while P2 inhibited *P. aeruginosa* biofilm. Surprisingly, all synthetic peptides having an elevated MIC on *C. albicans* planktonic cells, inhibited biofilm formation with an inhibition rate of about 40% for peptide P2 and between 20–30% for the others at the higher concentrations tested.

In short, peptides P1, P2, and P4 seemed to have the best inhibition activity also at concentrations of 40–60 μM for all microorganisms tested compared to compounds P0 and P3. This effect was unexpected, given the fact that peptide P0, similarly to the other analogues, only reduced planktonic cell growth and did not completely inhibit it.

**Figure 4:**
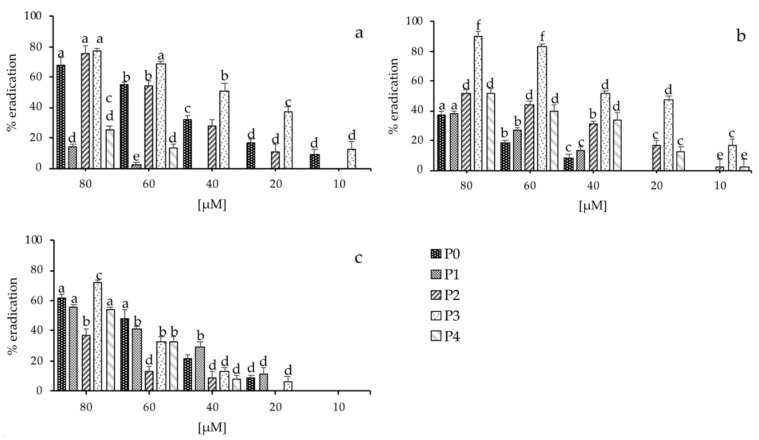
Eradication activity of the synthetized peptides. Comparison of minimal biofilm eradication concentration (MBEC) of the peptides P0, P1, P2, P3, and P4 on mature biofilms grown in 96-well polystyrene plates (**a**) *S. aureus*; (**b**) *P. aeruginosa*; (**c**) *C. albicans*. Error bars represent standard deviation. Error bars represent standard deviation. Different letters on top of each column (a–f) represent the significance at the 0.05 level (Tukey’s, *p* < 0.05).

The most demanding problem in antimicrobial research is to eradicate preformed biofilms. To quantify the effect of the five synthetic peptides toward established biofilms, we used the same sub-MIC concentrations between 10 to 80 μM for 24 h (Figure 4). Similar to the inhibition of biofilm formation by these peptides, the amount of viable *S. aureus*, *P. aeruginosa*, and *C. albicans* in the biofilms was reduced by these compounds in a dose-dependent manner. Furthermore, peptides did not only show eradication activity on bacterial biofilm, but also exhibited the biofilm dispersal effects on the *C. albicans* biofilm. Despite the improved activity of the analogues, complete eradication of sessile organisms within mature biofilms was not observed (Figure 4).

Significant dispersion (*p* < 0.05) of dose-dependent clearance of biofilm biomass was observed for peptides P0, P2, and P3, with the values of about 70%, 90%, and 80%, respectively (Figure 4). The weakest antibiofilm activity was documented only for peptide P1. In conclusion, preliminary antibiofilm experiments showed that peptide P2 seemed to have the best activity both in inhibition and eradication of biofilm of all three microorganisms tested. Although the relatively high peptide concentrations used are not fit for in vivo applications, they may be used for removal of biofilm in vitro.

## 3. Discussion

Octopus has developed a successful strategy for surviving in different hostile environments; octopus suckers continuously provide a great inspiration to biologists, engineers, and movie special effect supervisors for the development of novel bioinspired artificial devices [75,76,77,78]. Octopus arm suckers are specialized chemo-tactile organs with high sensitivity, equipped with millions of distributed sensory receptors allowing the animal to process in parallel massive amounts of mechanical and chemical information resulting from its densely innervation [16,21,22,79,80,81]. In fact, the octopus uses suckers for a variety of tasks, such as anchoring to the substratum, catching prey, locomotion, clean maneuvers, recognition by chemoreception, behavioral displays, and as a manipulating tool for collecting objects [82,83]. Octopus suckers are made of a tightly packed three-dimensional array of (radial, circular, and meridional) muscles with different fiber orientations [84,85]. They also have fibrous connective tissue layers and crossed connective tissue fibers, fixed in the musculature.

Calponin is a key protein involved in octopus muscular contraction and a large number of molecular isoforms were identified in *O. bimaculoides* (XP_014789315). Proteolytic modification of calponin seems to play a crucial role during the inflammatory response, but is also associated with rapid growth in octopus and other cephalopods, as result of an enhanced proteolytic activity present in animal fibers [86,87]. The maximum autolytic activity in octopus (*O. vulgaris*) arm muscle is 15-fold higher than in Pacific whiting, a fish well known to contain high levels of endogenous proteases [88], in particular cysteine and aspartic-proteinases, as cathepsin B [89]. Thus, it is believed that this proteolytic activity may be responsible of the release of antimicrobial peptides, which are exploited to enable the octopus to survive the harsh marine environment. Thus, it is reasonable to speculate that different proteolytic enzymes should be constitutively active in octopus fibers of suckers, generating protein fragments with antimicrobial activity (as observed in this study), similarly to what is observed in other animals in which actin-binding protein degradation products were demonstrated to play a role in various biological functions [90]. It is worth mentioning recent data regarding squid and cuttlefish demonstrating that AMPs trapped in the egg capsule confer efficient protection against microorganisms; those peptides are derived from female accessory sex glands, but also from the partial degradation of corresponding tissue proteins [24].

In this study, we isolated the pentapeptide AGTNK from *O. vulgaris* suckers and we proved that this molecule has a significant antimicrobial activity against *S. aureus* and *P. aeruginosa.* Sequence analysis demonstrated that this peptide is encrypted within the sequence of calponin-2-like isoform X1 and occurs therein multiple times (together with some variant sequences), yielding several bioactive peptide molecules from each parental protein, as result of the action of still-unknown proteases.

Starting from this preliminary data on the natural peptide OctoPatenopin, we designed and synthetized the C-terminal amidated homologue (P0) and four analogues with the aim of improving its antimicrobial performance. Synthetic peptides were analyzed for their antimicrobial activity against Gram-positive, Gram-negative bacteria, and yeast. The results clearly showed that the addition of one residue at the *N*-terminus did not induce any enhancement of activity. Conversely, peptide elongation at the C- and *N*-termini with short sequences present in the conserved, repeated motif of calponin-2-like isoform X1 (yielding peptides P2 and P3) was associated with a significant increase of corresponding antimicrobial activity. Interestingly, the whole repeated motif of calponin, as present in peptide P4, did not induce a significant enhancement of antimicrobial properties. All synthetic peptides were overall more active compared to the natural compound, pointing out the need of additional structure–activity functional studies to decipher the structural elements essential for activity. Further studies are also necessary to identify the most active sequence, which will probably comprise modifications both at the C- and *N*-terminus of the native sequence P0.

We also performed an in vitro experiment to assess peptide antibiofilm activity, analyzing both inhibitory effects on biofilm formation and dissolution on mature biofilm. To this purpose, we tested the peptides under or at MIC concentrations. Our results showed a peptide concentration-dependent inhibition of biofilm formation and a good eradication capacity for all microorganisms tested, suggesting a cell disaggregation and disruption mechanism [91,92,93,94]. In particular peptides P1, P2, and P4 seemed to have the best inhibition activity for all microorganisms tested, whereas significant eradication of dose-dependent clearance of biofilm biomass was observed for peptides P0, P2, and P3. In conclusion, preliminary antibiofilm experiments showed that peptide P2 seemed to have the best activity in both inhibition and eradication of biofilm of all three microorganisms tested.

Although peptides with moderate MIC values (between 50 and 200 μM) are not suitable candidates for use as single potent antibiotics in the pharmaceutical industry; nonetheless, they could find applications in synergy with conventional antibiotics, further favoring the entry of other drugs by destabilizing the microorganism membrane. At the same time, due to their natural origin and their presence in edible material, they may find promising applications in the food industry to increase shelf-life of food products through dedicated treatments and/or as active ingredients for packaging, as they do not cause harmful or undesirable side effects [65,95].

Tandem repeats in proteins is not a new phenomenon but is widely reported in literature. An interesting example is the presence in many organisms of pattern recognition receptors (known as PRRRs), which are part of the innate immune system, and are deputed to recognition and binding of conserved pathogen associated molecular patterns (known as PAMPs). These tandem repeats often present antibacterial activity when used as peptides [96]. It is likely that in the octopus suckers, tandem repeats play several roles and their eventual release into the medium may be critical for immunity similarly to what has been found for other AMPs present as tandem repeats in host proteins [97,98,99]. Clearly, we cannot exclude that the presence of tandem repeats in the suckers will also favor the formation of particular secondary structure motifs, which likely play a key role in the activity of the protein.

As a matter of fact, our results could be speculatively claimed to interpret octopus maternal care behavior, in which animal female broods and tends fertilized eggs until they hatch [100]. The chorion tissue of eggs allows the exchange of oxygen; thus, maternal care and mother movements were interpreted as essential for preventing embryo fouling and suffocation [101,102]. Based on the results presented in this study, female arm movements and egg touching through suckers should allow animal oxygenation and cleaning of fertilized eggs, but also corresponding protection from microorganism-driven infections [24]. Likely, octopus females may release antimicrobial peptides, such as OctoPartenopin, directly on the eggs to protect them from the attack of pathogens. In this context, it was previously demonstrated that when females abandon their eggs, the latter die [103]. Further studies are requested in this context to prove the functional role of OctoPartenopin and female animal suckers in egg protection from pathogens.

## 4. Materials and Methods

### 4.1. Octopus Collection

Adult specimens of *O. vulgaris (n* = 6, weight range 600–800 g) were captured in the Bay of Naples. Octopuses were transferred to the Department of Biology, as reported in Di Cosmo et al. [104], and were sacrificed as described in Polese et al. [105]. Animals were dissected in sterile conditions, isolating each single sucker. The experiments in the present study were conducted in accordance with the principles and procedures that were approved by the Institutional Animal Care of the University of Napoli Federico II and the Ministry of Health (project no. 608/2016-PR-17 June 2016; protocol no. DGSAF 0022292-P-3 October 2017), and according to the Italian and European law (European Directive 2010/63 EU L276; Italian DL. 4 March 2014, no. 26; the ethical principles of Reduction, Refinement, and Replacement).

### 4.2. Methanolic Extraction of Peptides from Octopus Suckers

Octopus suckers were dissected, and methanol was added to tissue (800 g of tissue in 1.5 L of methanol). The mixture was agitated in an orbital shaker for 15 days at room temperature (RT). Subsequently, tissue was removed, and the mixture was centrifuged (10,000× *g*, 5 min, at RT) to collect the supernatant, which was filtered through 0.45 µm membranes, concentrated to 250 mL under vacuum and finally lyophilized. For extract delipidation, the dried material was dissolved in H_2_O (250 mL), added with an equal volume of hexane, stirred for 5 min and finally centrifuged (10,000× *g*, 5 min, at RT). The resulting supernatant was then discarded, while the remaining aqueous solution (100 mL) was used for subsequent analysis.

### 4.3. Bacterial Strain, Media, and Culture Conditions

Microorganisms used for this study were as follows: *Staphylococcus aureus* ATCC 6538, *Pseudomonas aeruginosa* ATCC 9027, and *Candida albicans* ATCC 90028. Microorganisms were routinely cultured in tryptic soy broth (TSB) supplemented with 0.1% glucose, at 37 °C, for 18 h prior to experiments, and corresponding cell concentration was adjusted to 10^6^ CFU mL^−1^ by optical density at 600 nm. Stock cultures were maintained in trypticase soy agar (TSA) and Sabouraud dextrose agar containing glycerol at −80 °C.

### 4.4. Antimicrobial Agar Diffusion Assay of the Extract

For antimicrobial testing, the agar disc diffusion method was used and the antimicrobial susceptibility was detected by analyzing the inhibition zones, measured and compared according to the standards set by the CLSI [106], which classify the strains as sensitive (S), intermediate (I), or resistant (R). All tests were performed in duplicate, and the antimicrobial activity was expressed as the mean of inhibition diameters (mm, ±standard deviation, SD) produced by the extracts.

Microorganisms were cultivated overnight and a suspension containing 10^6^ CFU mL^−1^ was spread on plates containing TSA or Sabouraud agar. Holes of approximately 5 × 3 mm were made in the agar and were filled with 30 μL of the extract stock solution (1 mg/mL). After incubation at 37 °C for 24 h, inhibition zones were measured in millimeters and compared to the controls. Inhibition halos ≥6 mm were considered as evidence of antimicrobial activity. For each strain, the negative control was a hole filled only with solvent, and the positive control was a hole with 5 μg ampicillin (AMP), 5 μg gentamicin (G), and 1 μg amphotericin B (AMPH-B).

### 4.5. Sucker Extract (SE) Purification and Characterization

The aqueous solution of SE reported above was assayed for protein concentration, which was determined with the Bradford assay (Bio-Rad Protein Assay Kit 5000001). Then, aliquots of this aqueous solution of SE (500 μL) were initially resolved by explorative semi-preparative chromatography on a VP 250/10 Nucleodur 300-5 C18 column (Macherey-Nagel GmbH, Düren Germany) connected to a 1260 Infinity II LC system (Agilent Technologies, Santa Clara, CA, USA), which allowed a step-gradient elution of solvent B (acetonitrile containing 0.1% trifluoroacetic acid - TFA) in solvent A (aqueous 0.1% TFA), at a flow rate of 3.5 mL/min. The elution was achieved using the following conditions: isocratic elution with 2% B for 5 min, which was followed by the subsequent gradient steps: (i) from 2% to 5% B over 5 min; (ii) from 5% to 20% B over 5 min; (iii) from 20% to 40% B over 20 min; (iv) from 40% to 95% B over 10 min. The elution absorbance was monitored at 220 nm. An automatic fraction collector was set in a time-based mode in order to collect one fraction every 6.5 min (starting from min 1). Three semi-preparative runs were performed consecutively with the aim to increase the amount of purified material available for subsequent analyses; similar fractions from different runs were pooled together.

In order to optimize the recovery of bioactive molecules, a second experiment was performed on the same chromatographic system (instrument, column and solvents) reported above. In this case, 50 μL-aliquots of SE were injected. The initial chromatographic flow rate was set at 3.5 mL/min; at 3.1 min, the flow rate was lowered to 1.0 mL/min, and was maintained constant until the end of the chromatography. Elution was achieved using the following conditions: isocratic elution with 2% B for 12 min, which was followed by the subsequent gradient steps: (i) from 2% to 5% B over 0.1 min; (ii) from 5% to 20% B over 3 min; (iii) from 20% to 40% B over 20 min; (iv) from 40% to 95% B over 10 min. The automatic fraction collector was set in a peak-based mode. Six semi-preparative runs were performed consecutively with the aim to increase the amount of purified material; based on the chromatographic profile, similar fractions from different runs were pooled together to get enough purified material for subsequent functional and structural characterization. In all cases reported above, chromatographic fractions were assayed for antimicrobial activity as described below.

### 4.6. Peptide Characterization of Extract Fractions

NanoLC-ESI-Q-Orbitrap MS/MS analysis was performed using a LTQ XL Q-ExactivePlus mass spectrometer equipped with a Nanoflex ion source (ThermoScientific, Waltham, MA, USA) and connected to an UltiMate 3000 HPLC RSLC nano system-Dionex (ThermoScientific, Waltham, MA, USA). Peptides were separated on an Acclaim PepMap RSLC C18 column, 150 mm × 75 μm i.d., 2-μm particles, 100-Å pore size (ThermoScientific) as previously reported [107]. Full mass spectra were acquired in the range *m*/*z* 200–1000, with nominal resolution 70,000. Fragmentation of parent ions was controlled by a data-dependent scanning procedure over the most abundant ions using 20 s dynamic exclusion. Mass isolation window and collision energy were set to *m*/*z* 1.2 and 30%, respectively. Raw data were searched with PEAKS Studio 8.0 (Bioinformatics Solutions Inc., Waterloo, ON, Canada) against a database containing octopus protein sequences retrieved from NCBI (64842 sequences). Searching parameters were no enzyme, Met oxidation, *N*-terminal Gln and Glu cyclization, Asn/Gln deamidation, as variable modifications. Mass tolerance values for peptide matches were set to 10 ppm and 0.05 Da for precursor and fragment ions, respectively. The threshold value for PEAK peptide identification score (−10logP) was set to 23, corresponding to a false discovery rate (FDR) <0.1%. In parallel, automated de novo sequencing routines were applied to raw mass spectrometric data using PEAKS; in this case, a threshold value for average local confidence (ALC) of 90% was used. Peptide identity assignment was always validated by manual interpretation of the corresponding MS/MS spectra.

### 4.7. Peptide Synthesis and Purification

Peptide analogues were synthetized by the standard solid phase fluorenylmethyloxycarbonyl (Fmoc) amino acid method. The rink amide resin p-methylbenzhydrylamine (MBHA) was used (substitution: 0.5 mmol/g) and the synthesis was performed on a scale of 100 μmol. After coupling of each amino acid, the amino group was deprotected and the process was repeated to get the desired peptide sequence, the protocol is reported below [108]. The first coupling was carried out in the presence of 4 equiv of Fmoc- amino acid, 4 equiv *N,N*′-Diisopropylcarbodiimide (DIC), and 4 equiv ethyl cyano(hydroxyimino)acetate (oxymapure) for 30 min; all the other couplings were carried out in the presence of 4 equiv Fmoc-amino acid, 4 equiv 1-[bis(dimethylamino)methylene]-1H-1,2,3-triazolo[4-5-b]pyridinium 3-oxide exafluorophosphate (HATU), and 8 equiv *N,N*′-di-isopropylethylamine (DIPEA) for 30 min. The Fmoc removal was performed with 30% (v/v) piperidine in *NN*-dimethylformamide for 10 min. The crude peptides were cleaved from the resin with an acid solution composed by 95% of TFA in presence of scavengers if required (ethane dithiol, triisopropyl silane) and after precipitated with ice cold ethyl ether. The obtained peptides were purified by preparative reverse-phase HPLC on a Waters Delta-Prep 3000 chromatography system equipped with a UV Lambda Max Model 481 detector. The samples were eluted with a linear gradient (from 5 to 70% in 20 min) of solvent B (acetonitrile containing 0.1% TFA) in solvent A (aqueous 0.1% TFA), at a flow rate of 20 mL/min. The peptide purity and identity were checked with mass spectrometry analysis with a LTQ-XL mass spectrometer (ThermoScientific, Waltham, MA, USA).

### 4.8. Antimicrobial Assay (MIC Determination) of the Purified Fraction and Synthetic Peptides

The minimum inhibitory concentration (MIC) determination was conducted as described in our previous papers [94,109]. Briefly, microbes were inoculated in TSB and incubated at 37 °C to the exponential phase. Then, the inoculum was diluted with fresh TSB to 10^6^ CFU/mL, and 50 μL microbial dilutions were mixed with serial dilutions of fractions or peptides (50 μL) in 96-well microtiter plates. The plates were incubated at 37 °C for 18 h, and the minimum concentrations at which no visible growth of microorganisms occurred were recorded as MIC values according to the methods described by the Clinical and Laboratory Standards Institute [106].

### 4.9. Biofilm Inhibition and Eradication Assay

The microorganism’s ability to form biofilm was determined using the method previously described in our laboratory [94]. Bacterial and fungal biofilm prevention and eradication assays were performed based on our published protocols. Synthetized peptides (P0–P4) were tested at sub-MIC concentrations ranging from 10 to 80 μM in triplicate in both assays. For the prevention of biofilm formation, diluted cultures of each strain were added to 96-well plates and incubated with peptides (at concentrations ranging from 10 to 80 μM) at 30 °C, for 24 h. For the eradication of mature biofilm, diluted cultures of *C. albicans* and two bacteria were added to 96-well plates and incubated at 30 °C. After 24 h, the planktonic cells and medium were aspirated from the wells and fresh medium was added to the well, followed by peptides at the same concentrations. Plates were incubated at 30 °C, for an additional 24 h. For both above mentioned assays, the planktonic cells and medium were aspirated at the end of the final incubation period, and wells were washed twice with PBS. The residual attached biofilms were fixed with 95% v/v ethanol per well and, after 20 min, plates were emptied and left to dry. Plates were stained for 15 min with 0.1% w/v crystal violet. Excess stain was then removed by rinsing the plate with PBS. The plates were allowed to dry, and the crystal violet-stained biofilm was solubilized with 200 μL of 30% v/v glacial acetic acid per well. The OD of each well was measured at 550 nm with a microplate reader [110]. The same formula described above was used to quantify the biofilm biomass forming when the incubation was done with peptides or when remaining after incubation with peptides. The percentage of biofilm inhibition and eradication was determined by the formula: biofilm reduction % = OD control − OD sample/OD control × 100%.

### 4.10. Statistical Analysis

Statistical analyses were performed using Microsoft^®^ Excel 2016/XLSTAT©-Pro (version 7.2, Addinsoft, Inc., Brooklyn, NY, USA). Values were expressed as the mean ± standard deviation (±SD). Data were assessed considering the analysis of variance (ANOVA) and Tukey’s test to check any difference among the groups (*p* < 0.05).

## Figures and Tables

**Table 1:** Antimicrobial resistance of sucker extract and HPLC fractions. Disk diffusion zone diameter (mean ± standard deviation, SD; mm) interpretative chart of sucker extract (SE) and different HPLC fractions (from A to F) against *S. aureus*, *P. aeruginosa*, and *C. albicans*. AMP: Ampicillin; G: Gentamicin; AMPH-B: amphotericin B; R: Resistant; I: Intermediate; S: Sensitive; NT: not-tested.

Strain	SE	HPLC Fraction						Positive Control		
		A	B	C	D	E	F	AMP	G	AMPH-B
Mean ± SD (Ø mm)										
**Gram-positive**										
*S. aureus* ATCC 6538	11.0 ± 1.2	12.0 ± 2.0	-	8.0 ± 1.5	-	6.0 ± 2.8	-	S	S	NT
**Gram-negative**										
*P. aeruginosa* ATCC 9027	8.0 ± 2.3	10.0 ± 3.1	-	6.0 ± 3.0	-	-	-	R	S	NT
**Yeast**										
*C. albicans* ATCC 90028	-	7.0 ± 1.8	-	-	-	-	-	NT	NT	S

**Table 2:** MIC of low flow HPLC fractions. Minimal inhibitory concentration (MIC) required for 80% inhibition of cell growth in liquid medium of the HPLC fraction derived from low-flow reverse phase C18-HPLC separation of delipidated methanolic extract of octopus sucker (SE) (Figure 1b); these results represent mean values of three replicates ± standard deviation, SD.

Strain	MIC80 (µg/mL) HPLC Fraction					
	A1	A2	A3	A4	A5	A6
*S. aureus* ATCC 6538	150 ± 1	120 ± 5	180 ± 4	120 ± 2	120 ± 5	200 ± 6
*P. aeruginosa* ATCC 9027	>300	200 ± 2	>300	180 ± 5	>300	200 ± 5
*C. albicans* ATCC 90028	>300	>300	>300	300 ± 2	>300	>300

**Table 3:** MIC of the synthetized peptides. Minimal inhibitory concentration required for 80% inhibition of cell growth in liquid medium; results represent mean values of three replicates ± standard deviation, SD.

Sequence			MIC80 (µM)		
		Mass Value	*S. aureus* ATCC 6538	*P. aeruginosa* ATCC 9027	*C. albicans* ATCC 90028
P0	NH_2_-AGTNK-CONH_2_	488.53	150 ± 2	200 ± 3	200 ± 3
P1	NH_2_-QAGTNK-CONH_2_	616.66	150 ± 3	100 ± 5	200 ± 5
P2	NH_2_-QAGSNKGASQKGMS-CONH_2_	1349.47	50 ± 5	50 ± 2	100 ± 3
P3	NH_2_-EGQGVISLQAGTNK-CONH_2_	1400.54	80 ± 1	50 ± 3	100 ± 4
P4	NH_2_-GEGIIGLQAGTNKFASQIGMKIGAVRHIADIR-CONH_2_	3321.88	80 ± 5	50 ± 2	180 ± 5

## Supplementary Materials

The following are available online at https://www.mdpi.com/1660-3397/18/8/380/s1, Table S1: Detailed results of *De novo* sequencing and Database searching analysis of nanoLS-ESI-MS/MS raw data acquired for Fraction A4.
