# Supplementary material for: OctoPartenopin: Identification and Preliminary Characterization of a Novel Antimicrobial Peptide from the Suckers of Octopus vulgaris
Source: Mar Drugs. 2020 Jul 23;18(8):380. doi: 10.3390/md18080380 (PMC7460285; doi:10.3390/md18080380)
Supplement: Supplementary file 1 [file marinedrugs-18-00380-s001.pdf]

Supplementary Table S1: Detailed results of *De novo s* equencing and Database searching analysis of nanoLS-ESI-MS/MS raw data acquired for Fraction A4.

Peptide "*De novo* sequencing"

Peptide sequence	Tag Length	ALC (%)	length	ppm	m/z	z	RT	Area	Mass	local confidence (%)
AGTNK	5	97	5	2.6	245.635	2	2.53	1.87E+08	489.2547	96 98 99 98 98
KAAK	4	94	4	3.4	209.145	2	1.83	8.53E+05	416.2747	94 96 94 94
SNVGK	5	93	5	2.3	252.643	2	2.02	1.00E+06	503.2704	97 92 92 93 94

Peptide identification by "Database Search" approach

Peptide sequence	-10lgP	Mass	Length	ppm	m/z	RT	Area	Scan	#Spec	Accession number from NCBI protein databse
AGTNK	27.17	489.255	5	2.6	245.635	2.53	1.87E+08	829	35	gi|918281088:gi|961140629
KPKPAH	27.1	676.402	6	9.6	226.477	2.85	ND	928	1	gi|918300745
SGGVGK	25.01	503.27	6	2.4	252.643	2.02	1.00E+06	659	1	gi|961111469
EAKVG	24.12	502.275	5	-0.2	252.145	3.69	4.15E+06	1187	1	gi|918336563:gi|918334154:gi|961075932:gi|918302746:gi|961118027:gi|961118024:gi|918302745:gi|961118030
QSGKAK	23.67	617.35	6	2.3	309.683	3.69	5.40E+05	1188	1	gi|961108556:gi|918309491:gi|918301884:gi|918301885:gi|961119206
SAGGK	23.56	418.218	5	2.6	210.117	1.89	2.46E+06	614	2	gi|918337123:gi|918315619:gi|918303805:gi|918303804
SATNK	23.28	519.265	5	2.4	260.641	2.02	1.66E+06	658	1	gi|918335123:gi|918286505:gi|918315892:gi|918304868:gi|918304867:gi|918339210
